# Associations of poor sleep quality, chronic pain and depressive symptoms with frailty in older patients: is there a sex difference?

**DOI:** 10.1186/s12877-022-03572-9

**Published:** 2022-11-16

**Authors:** Shanshan Shen, Xingkun Zeng, Yinghong Yang, Huilan Guan, Lingyan Chen, Xujiao Chen

**Affiliations:** Department of Geriatrics, Zhejiang Hospital, No. 12 Lingyin Road, Hangzhou, 310013 People’s Republic of China

**Keywords:** Poor sleep quality, Chronic pain, Depression, Frailty, Older adult, Sex differences

## Abstract

**Background:**

Sleep disturbance, chronic pain and depressive symptoms later in life are modifiable risk factors and may contribute to frailty. However, much less is known about sex differences in the association between these concurrent symptoms and frailty in older patients. Therefore, we conducted this study to explore the associations of poor sleep quality, chronic pain, and depressive symptoms with frailty in older patients, and the sex-specific associations.

**Methods:**

In an observational population-based study, 540 older hospitalized patients from Zhejiang Hospital in China were enrolled. We collected data on poor sleep quality, pain, depressive symptoms and frailty using the Pittsburgh Sleep Quality Index, the Numerical Rating Scale, the 15-item Geriatric Depression Scale, and the Clinical Frailty Scale. Multivariate logistic regression models were used to explore the total sample and sex-specific associations among symptom burdens, symptom combination patterns and symptom counts, and frailty.

**Results:**

After adjusting for the potential covariates, concurrent poor sleep quality and depressive symptoms (OR = 4.02, 95% CI 1.57–10.26), concurrent poor sleep quality and chronic pain (OR = 2.05, 95% CI 1.04–4.05), and having three symptoms (OR = 3.52, 95% CI 1.19–10.44) were associated with a higher likelihood of frailty in older inpatients. In addition, older patients with 2 or 3 symptoms (2 and 3 vs. 0 symptoms) had a higher risk of frailty, and the odds ratios were 2.40 and 3.51, respectively. Interaction analysis and sex-stratified associations exhibited conflicting results. The nonsignificant effect of the interaction of sex and symptoms on frailty, but not the sex-stratified associations, showed that individual symptoms, symptom combination patterns, and symptom counts were associated with elevated risks of frailty in older male patients, but not in older female patients.

**Conclusions:**

Increased symptom burdens were associated with a higher risk of frailty in older inpatients, especially in those with poor sleep quality concurrent with at least one of the other two symptoms. Thus, a multidisciplinary program addressing these common symptoms is required to reduce adverse outcomes.

**Supplementary Information:**

The online version contains supplementary material available at 10.1186/s12877-022-03572-9.

## Background

Frailty is described as a clinically recognizable syndrome that increases multiple system vulnerability due to minor stressors, resulting in decreased physiological reserves and a decline in the ability to maintain homeostasis [1–3]. It is known to impose negative effects on health outcomes, such as disability, hospitalization, institutionalization and death [2, 4, 5]. In China, the overall prevalence of frailty in community-dwelling older adults is approximately 10%, and between 18 and 54% of cases of frailty among hospitalized patients are based on various diagnosed approaches [6–12]. Identifying modifiable risk factors for frailty and developing targeted screening and preventive and therapeutic interventions for older patients are of particular importance.

Older patients often experience multiple simultaneous physical and psychological symptoms due to multiple comorbidities and treatments. Sleep disturbance, chronic pain and depressive symptoms later in life are common symptoms, and can be treatable. Individual symptoms have been reported to be associated with frailty in older adults. For example, sleep disturbances, including poor sleep quality, reduced sleep efficiency, prolonged sleep latency, prolonged or short sleep duration and sleep-disordered breathing are associated with the prevalence and incidence of frailty in the elderly population [13–16]. Similarly, chronic pain was associated with an increased risk and severity of frailty in older individuals, especially increased experiences of exhaustion and low physical activity levels [17–19]. Additionally, several studies have shown that depressive symptoms increase the risk of incident frailty, and decrease the likelihood of reversal in frail older adults [20]. Recently, one study from the National Health and Aging Trend Study (NHATS) has focused on the cumulative association of pain, insomnia (difficulty initiating sleep and difficulty maintaining sleep) and depressive symptoms with frailty among older adults. The results showed that pain, difficulty initiating sleep and depressive symptoms had a synergistic effect on frailty, and the odds ratio increased to 10.20-times than that of individual symptoms [21]. In addition, significant sex differences have been reported in insomnia subtypes and frailty, but a synergistic effect of the mentioned symptoms on frailty has not been reported [22]. Specifically, older males with difficulty maintaining sleep and older females with difficulty initiating sleep were more prone to pre-frailty and frailty than those without insomnia symptoms [22]. Other existing studies also supported that sex differences in the association between other insomnia parameters and frailty [16, 23–25], between pain and frailty [17], and between depressive symptoms and frailty [26]. In addition, sex disparity in frailty and the health-mortality paradox have become increasingly concerning. In contrast to males of the same age, females had a higher prevalence and experienced a greater level of frailty, but often showed a lower risk of mortality [27, 28]. Thus, the evidence of sex differences between multiple symptoms and frailty warrants further investigation in clinical settings.

Given the high prevalence and reversibility of frailty, we conducted this study to explore the association of poor sleep quality, chronic pain and depressive symptoms with frailty in the older inpatients, and the sex-specific associations, in order to facilitate risk stratification and clinical decision-making for patients with frailty.

## Methods

### Study population

This study was designed as a cross-sectional study including 540 older patients (from October 2014 to September 2018) hospitalized in the geriatric department of Zhejiang Hospital, and all the assessments were performed when the patients were in a relatively stable condition during the hospitalization. All participants who were aged 60 years and older, and able to understand and communicate in Chinese were enrolled in the study. Participants with a history of severe dementia, a Mini-Mental State Examination (MMSE) score less than 18, clinically diagnosed mental diseases such as depressive disorders or anxiety disorders and antidepressants use, and acute pain were excluded. Patients who were unable to complete pain, depression, poor sleep quality and frailty assessments for a variety of reasons were also excluded. This study was approved by the medical ethics committees of Zhejiang Hospital, and all the participants provided written informed consent to use their clinical records. The design of the study and conducted procedures conformed to the ethical principles of the Helsinki Declaration.

### Symptom burden assessment

Symptom burden assessment included symptom combination patterns and symptom counts of poor sleep quality, chronic pain and depression.

The Pittsburgh Sleep Quality Index (PSQI) is a widely used, self-report questionnaire that assesses poor sleep quality in the last month [29, 30]. It consists of seven components: subjective sleep quality, sleep latency, sleep duration, sleep efficiency, sleep disturbance, sleep medication usage and daytime dysfunction. The score of each component ranges from 0 to 3, and the total score ranges from 0 to 21. A score greater than 5 indicated poor sleep quality in this study [31].

Chronic pain was defined as pain lasting for 3 months or more in one person [32]. The following question was asked: “In the last six months, have you experienced pain in any part of your body that lasted or recurred for more than 3 months?”. We used the Numerical Rating Scale (NRS) to assess pain severity during the previous week, with scores ranging from 0 (no pain) to 10 (worst imaginable pain) [33]. A score of 1 or above was defined as the presence of pain in this study.

Depressive symptoms were measured using the 15-item Geriatric Depression Scale (GDS-15). The total GDS-15 score ranges from 0 to 15, and a score of ≥ 6 is defined as the presence of depressive symptoms [34]. This cut-off value is insufficient for the diagnosis of depression, but it suggests that the severity of depressive symptoms requires further professional assessment and management [35].

### Frailty assessment

Frailty was assessed by the Clinical Frailty Scale (CFS), which depends on the clinicians’ judgements in terms of mobility and independent ability in daily life [36]. The total score ranges from 1 (very fit) to 7 (severely frail). A CFS score of ≥ 5 indicates frailty [37], and this cut-off showed a sensitivity of 94.1% and a specificity of 85.2% for older Chinese hospitalized patients [38].

### Other covariates

Demographic variables, including age, sex, educational level, marital status, cigarette smoking, alcohol use, main diagnosis on admission, concurrent chronic diseases, medication usage and time from admission to assessment, were collected. Body mass index (BMI) was calculated by height and weight. The comorbidity burden was assessed using the Cumulative Illness Rating Scale for Geriatrics (CIRS-G) [39]. It includes 14 systemic diseases, with severity assessed on a scale of 0 to 4. Higher CIRS-G scores indicate higher comorbidity. Polypharmacy was considered the concomitant use of ≥ 5 medications [40]. Cognitive function was measured using the Chinese version of the MMSE [41], and a higher MMSE score indicated good cognitive function.

### Statistical analysis

Data were analysed using SPSS 18.0 software (SPSS, Chicago, IL, USA). Normally distributed continuous variables are presented as the means ± standard deviations (SDs). Abnormally distributed continuous variables are presented as medians and inter-quartile ranges (IQRs), and categorical variables are expressed as numbers (percentages). Differences between groups among clinical characteristics and frailty status between groups were analysed using the unpaired *t test* and the chi-square test if necessary and the Mann–Whitney *U*-*test* where appropriate. To explore the impact of individual symptoms, symptom combination patterns and symptom counts on frailty in the total sample, we created a series of multivariate logistic regression models. Model 1 was crude model with no variable adjustments; Model 2 was adjusted for age, marital status, educational level, CIRS score, polypharmacy, time from admission to assessment and MMSE score, and Model 3 was adjusted for all the covariates in the Model 2 plus the interaction of sex and symptoms. Furthermore, we explored the sex-stratified associations of individual symptoms, symptom combination patterns, counts, and frailty using multivariate logistic regression models. Odds ratios (ORs) and 95% confidence intervals (CIs) are presented for the models. In addition, multicollinearity was analysed using the variance inflation factor (VIF), and no significance was found (VIF < 10). A *P* value of < 0.05 was defined as statistically significant.

## Results

### Enrolment, prevalence rate and characteristics in the total sample

This study included 924 older patients who agreed to participate. Patients who lacked important variables for three symptoms and frailty status (*n* = 174), as well as those who had a history of dementia (*n* = 17), an MMSE score < 18 (*n* = 100), a clinically diagnosed depressive or anxiety disorder (*n* = 31), or acute pain (*n* = 62), were excluded. The general characteristic differences between the included patients and excluded patients are presented in Table S1.

**Fig. 1:**
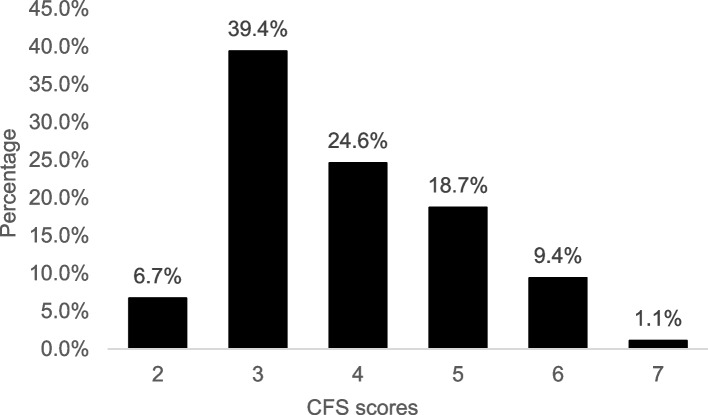
The distribution of CFS scores

**Fig. 2:**
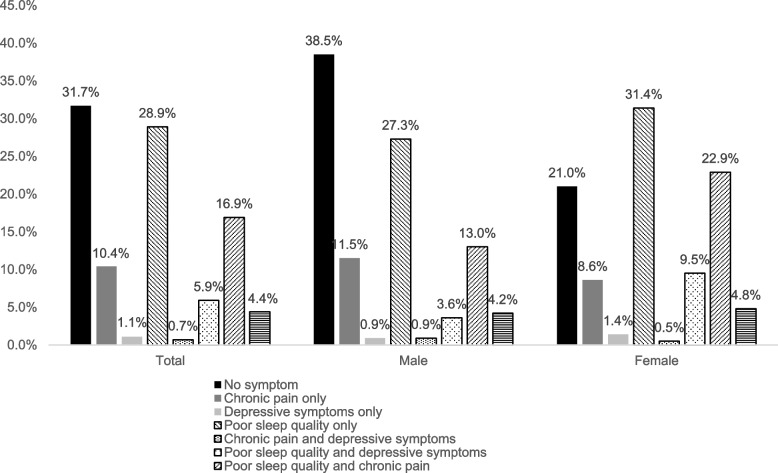
The prevalence of poor sleep quality, chronic pain, and depressive symptoms combinations of males and females

Of the 540 older patients in the final sample, 158 (29.3%) experienced frailty. Fig 1 shows the distribution of CFS scores. The prevalence rates of poor sleep quality, chronic pain, and depressive symptoms were 55.9%, 32.6% and 12.2%, respectively. The two most overlapping symptoms were poor sleep quality and chronic pain, with a prevalence of 16.9%, and the overlapping prevalence of the three coexisting symptoms was 4.4%. In addition, approximately 70% of patients experienced at least one symptom, and 30% experienced at least two symptoms. Fig 2 also shows the three symptom combination patterns of males and females.

### General characteristics between males and females

As shown in Table 1, males were older, more exposure to cigarette smoking and alcohol use, had higher education levels and had higher rates of polypharmacy than females (all *P* < 0.05). However, female patients were more likely to be widowed or divorced, experienced poorer sleep quality and had more depressive symptoms than males (all *P* < 0.05). Additionally, a significant difference in disease diagnosis on admission was observed in males and females (*P* < 0.05).

**Table 1:** General characteristics between male and female inpatients

	Total (*n* = 540)	Male (*n* = 330)	Female(*n* = 210)	*P-*value
Age (mean ± SD, years)	78.51 ± 8.07	79.50 ± 7.96	76.94 ± 8.02	** < 0.001**
Age ≥ 80 years, n (%)	280(51.9)	185(56.1)	95(45.2)	**0.014**
Educational level, n (%)				**0.026**
≤ 6 years	96(17.8)	49(14.8)	47(22.4)	
≥ 7 years	444(82.2)	281(85.2)	163(77.6)	
Widowed or divorced, n (%)	127(23.5)	59(17.9)	68(32.4)	** < 0.001**
Current or former smoker, n (%)	136(25.2)	132(40.0)	4(1.9)	** < 0.001**
Current or former drinker, n (%)	132(24.4)	124(37.6)	8(3.8)	** < 0.001**
BMI (mean ± SD, kg/m^2^)	23.78 ± 3.36	23.84 ± 3.15	23.68 ± 3.66	0.594
Main diagnosis on admission, n (%)				** < 0.001**
Cardiovascular diseases	113(20.9)	70(21.2)	43(20.5)	
Peripheral vascular diseases	222(41.1)	134(40.6)	88(41.9)	
Nervous system diseases	48(8.9)	25(7.6)	23(11.0)	
Respiratory diseases	67(12.4)	56(17.0)	11(5.2)	
Other	90(16.7)	45(13.6)	45(21.4)	
CIRS-G [median (IQR), scores]	9(7,12)	9(7,13)	9(7,11)	0.085
Polypharmacy (≥ 5 drugs), n (%)	264(48.9)	173(52.4)	91(43.3)	**0.039**
Time from admission to assessment [median (IQR), days]	0(0,2)	0(0,2.25)	0(0,2)	0.102
MMSE (mean ± SD, scores)	26.04 ± 3.02	26.02 ± 2.86	26.07 ± 3.27	0.838
PSQI [median (IQR), scores]	6(3,10)	5(3,9)	8(5,11)	** < 0.001**
Poor sleep quality, n (%)	302(55.9)	159(48.2)	143(68.1)	** < 0.001**
NRS [median (IQR), scores]	0(0,2)	0(0,2)	0(0,3)	**0.025**
Chronic pain, n (%)	176(32.6)	98(29.7)	78(37.1)	0.072
GDS-15 [median (IQR), scores]	2(1,4)	1(0,3)	2(1,4)	** < 0.001**
Depressive symptoms, n (%)	66(12.2)	32(9.7)	34(16.2)	**0.025**
Frailty, n (%)	158(29.3)	103(31.2)	55(26.2)	0.211

*BMI* body mass index, *CIRS-G* Cumulative Illness Rating Scale for Geriatrics, *MMSE* Mini-Mental State Examination, *PSQI* Pittsburgh Sleep Quality Index,

*NRS* Numerical Rating Scale, *GDS-15* 15-item Geriatric Depression Scale, *SD* standard deviation, *IQR* interquartile range. Significance difference *P* < 0.05 is

shown in bold

### General characteristics by sex and frailty status

Compared with nonfrail male patients, frail male patients were older, more likely to be widowed or divorced, had higher percentages of comorbidity burden and polypharmacy, had a longer time from admission to assessment, had lower MMSE scores, and experienced higher symptom burden, such as chronic pain, poor sleep quality and depressive emotions (all *P* < 0.05). Among in older female patients, frail patients were older, had a higher BMI, had higher rates of comorbidity burden and polypharmacy, had a longer time from admission to assessment and had lower MMSE scores (all *P* < 0.05), but did not suffer from higher symptom burden than compared with those without frailty, as detailed in Table 2.

**Table 2:** General characteristics of older inpatients according to sex and frailty status

	Male (*n* = 330)			Female (*n* = 210)		
	Non-frail (*n* = 227)	Frail (*n* = 103)	*P-* value	Non-frail (*n* = 155)	Frail (*n* = 55)	*P-* value
Age (mean ± SD, years)	77.08 ± 7.61	84.83 ± 5.85	** < 0.001**	74.72 ± 7.40	83.20 ± 6.19	** < 0.001**
Age ≥ 80 years, n (%)	98(43.2)	87(84.5)	** < 0.001**	53(34.2)	42(76.4)	** < 0.001**
Educational level, n (%)			0.666			0.105
≤ 6 years	35(15.4)	14(13.6)		39(25.2)	8(14.5)	
≥ 7 years	192(84.6)	89(86.4)		116(74.8)	47(85.5)	
Widowed or divorced, n (%)	29(12.8)	30(29.1)	** < 0.001**	45(29.0)	23(41.8)	0.082
Current or former smoker, n (%)	88(38.8)	44(42.7)	0.497	1(0.6)	3(5.5)	0.095
Current or former drinker, n (%)	78(34.4)	46(44.7)	0.073	6(3.9)	2(3.6)	1.000
BMI (mean ± SD, kg/m^2^)	24.03 ± 3.02	23.41 ± 3.41	0.107	23.25 ± 3.18	24.87 ± 4.58	**0.018**
Main diagnosis on admission, n (%)			**0.016**			0.512
Cardiovascular diseases	45(19.8)	25(24.3)		29(18.7)	14(25.5)	
Peripheral vascular diseases	105(46.3)	29(28.2)		70(45.2)	18(32.7)	
Nervous system diseases	13(5.7)	12(11.7)		15(9.7)	8(14.5)	
Respiratory diseases	33(14.5)	23(22.3)		8(5.2)	3(5.5)	
Other	31(13.7)	14(13.6)		33(21.3)	12(21.8)	
CIRS-G [median (IQR), scores]	8(6,11)	11.5(8,15)	** < 0.001**	8(6,10)	11(9,12)	** < 0.001**
Polypharmacy (≥ 5 drugs), n (%)	95(41.9)	78(75.7)	** < 0.001**	57(36.8)	34(61.8)	**0.001**
Time from admission to assessment [median (IQR), days]	0(0,2)	1(0,4)	** < 0.001**	0(0,1)	1(0,4)	** < 0.001**
MMSE (mean ± SD, scores)	26.70 ± 2.51	24.50 ± 3.00	** < 0.001**	26.39 ± 3.12	25.18 ± 3.53	**0.018**
PSQI [median (IQR), scores]	5(3,8)	7(3,11)	**0.002**	7(4,10)	11(6,14)	** < 0.001**
Poor sleep quality, n (%)	98(43.2)	61(59.2)	**0.007**	100(64.5)	43(78.2)	0.062
NRS [median (IQR), scores]	0(0,0)	0(0,3)	** < 0.001**	0(0,2)	0(0,3)	0.301
Chronic pain, n (%)	55(24.2)	43(41.7)	**0.001**	56(36.1)	22(40.0)	0.610
GDS-15 [median (IQR), scores]	1(0,2)	2(1,5)	** < 0.001**	2(1,4)	3(2,5)	**0.008**
Depressive symptoms, n (%)	14(6.2)	18(17.5)	**0.001**	23(14.8)	11(20.0)	0.372

*BMI* body mass index, *CIRS-G* Cumulative Illness Rating Scale for Geriatrics, *MMSE* Mini-Mental State Examination, *PSQI* Pittsburgh Sleep Quality Index,

*NRS* Numerical Rating Scale, *GDS-15* 15-item Geriatric Depression Scale, *SD* standard deviation, *IQR* interquartile range. Significance difference *P* < 0.05 is

shown in bold

### Associations of individual symptoms, symptom combination patterns and symptom counts with frailty in the total sample

Table 3 presents the associations of individual symptoms, symptom combination patterns, symptom counts and frailty in the total sample. In Model 1, individual symptoms, symptom combination patterns (poor sleep quality only, poor sleep quality concurrent with the other two symptoms), and symptom counts (0, 1, 2 and 3) were associated with a higher risk of frailty. Model 2, adjusting for the potential covariates, revealed that depression (OR = 2.29, 95% CI 1.21–4.35) was associated with a higher likelihood of frailty, and chronic pain (OR = 1.53, 95% CI 0.97–2.42) and poor sleep quality (OR = 1.59, 95% CI 0.99–2.53) were marginally associated with frailty. For symptom combination patterns of chronic pain, depressive symptoms, and poor sleep quality, older patients who had concurrent poor sleep quality and depressive symptoms (OR = 4.02, 95% CI 1.57–10.26), concurrent poor sleep quality and chronic pain (OR = 2.05, 95% CI 1.04–4.05), and having three concurrent symptoms (OR = 3.52, 95% CI 1.19–10.44) had increased odds ratios of being frailty than those without any symptoms after adjustment for potential covariates. We further explored the associations between symptom counts and frailty. Significance was observed in the associations between increased symptom counts (2 and 3 vs. 0) and frailty in the total sample, and the odds ratios in the Model 2 were 2.40 and 3.51, respectively.

**Table 3:** Associations between individual symptom, symptoms combination patterns and counts on frailty in the total sample

	Model 1	*P*-value	Model 2	*P*-value	Model 3	*P*-value
	OR(95%CI)		OR(95%CI)		OR(95%CI)	
**Individual symptom**						
Chronic pain						
No	1.00	-	1.00	-	1.00	-
Yes	1.71(1.16,2.51)	**0.007**	1.53(0.97,2.42)	0.070	1.89(1.05,3.41)	**0.033**
Depressive symptoms						
No	1.00	-	1.00	-	1.00	-
Yes	2.10(1.24,3.55)	**0.006**	2.29(1.21,4.35)	**0.011**	3.11(1.24,7.82)	**0.016**
Poor sleep quality						
No	1.00	-	1.00	-	1.00	-
Yes	1.79(1.22,2.63)	**0.003**	1.59(0.99,2.53)	0.053	1.62(0.92,2.84)	0.094
**symptom combination patterns**						
No symptom	1.00	-	1.00	-	1.00	-
Chronic pain only	1.82(0.92,3.62)	0.086	1.86(0.83,4.16)	0.133	2.41(0.97,6.04)	0.060
depressive symptoms only	2.09(0.37,11.91)	0.406	1.69(0.24,12.13)	0.600	1.99(0.13,29.64)	0.618
Poor sleep quality only	1.70(1.01,2.84)	**0.044**	1.53(0.83,2.83)	0.177	1.48(0.70,3.13)	0.301
Chronic pain and depressive symptoms	1.39(0.14,13.83)	0.777	1.00(0.03,31.03)	0.999	1.24(0.03,45.94)	0.906
Poor sleep quality and depressive symptoms	3.25(1.47,7.20)	**0.004**	4.02(1.57,10.26)	**0.004**	6.20(1.42,27.03)	**0.015**
Poor sleep quality and chronic pain	2.49(1.41,4.41)	**0.002**	2.05(1.04,4.05)	**0.038**	2.22(0.94,5.24)	0.069
Poor sleep quality and chronic pain and depressive symptoms	4.18(1.73,10.14)	**0.002**	3.52(1.19,10.44)	**0.023**	4.79(1.17,19.53)	**0.029**
**Symptom counts**						
0	1.00	-	1.00	-	1.00	-
1	1.74(1.08,2.81)	**0.024**	1.61(0.92,2.84)	0.097	1.75(0.90,3.41)	0.099
2	2.63(1.56,4.43)	** < 0.001**	2.40(1.28,4.50)	** < 0.001**	2.66(1.21,5.86)	**0.015**
3	4.18(1.73,10.14)	**0.002**	3.51(1.19,10.34)	**0.023**	4.76(1.17,19.35)	**0.029**

Model 1: no adjusted model

Model 2: adjusting for age, sex, marriage status, educational level, CIRS-G score, polypharmacy, time from admission to assessment and MMSE score

Model 3: adjusting for the covariates in the model 2 plus the interaction of sex and symptoms

*OR* odd ratio, *CI* confidence interval

Significance difference *P* < *0.05* is shown in bold

### Interaction analysis and sex-stratified associations of individual symptoms, symptom combination patterns and symptom counts with frailty

Model 3, as shown in Table 3, showed that significant differences were observed among individual symptoms, symptom combination patterns (poor sleep quality and depressive symptoms, poor sleep quality concurrent with the other two symptoms), and symptom counts (2 and 3 vs. 0) were associated with increased odds of being frailty. Nevertheless, no statistical differences were observed between the interaction of sex and symptoms and frailty in the total sample. In addition, sex-stratified analysis for males and females was performed in order to explore the possible associations. The results in Table 4 show that concurrent poor sleep quality and depressive symptoms (OR = 6.92, 95% CI 1.48–32.42), concurrent poor sleep quality and chronic pain (OR = 2.48, 95% CI 1.02–5.98), and having three concurrent symptoms (OR = 4.50, 95% CI 1.08–18.73) were associated with an elevated risks of frailty in older male patients. Older male patients with 2 or 3 symptoms (2 and 3 vs. 0) had a greater risk of frailty, and the odds ratios were 2.90 and 4.46, respectively. No significance was observed in female patients.

**Table 4:** Sex stratified associations between individual symptom, symptoms combination patterns and counts on frailty

	Male				Female			
	Model 1	*P*- value	Model 2	*P*- value	Model 1	*P*- value	Model 2	*P*- value
	OR(95%CI)		OR(95%CI)		OR(95%CI)		OR(95%CI)	
**Individual symptom**								
Chronic pain								
No	1.00	**-**	1.00	**-**	1.00	**-**	1.00	**-**
Yes	2.24(1.37,3.68)	**0.001**	2.03(1.11,3.71)	**0.021**	1.18(0.63,2.22)	0.610	1.07(0.51,2.25)	0.865
Depressive symptoms								
No	1.00	**-**	1.00	**-**	1.00	**-**	1.00	**-**
Yes	3.22(1.53,6.77)	**0.002**	2.98(1.16,7,70)	**0.024**	1.44(0.65,3.18)	0.374	1.75(0.71,4.32)	0.222
Poor sleep quality								
No	1.00	**-**	1.00	**-**	1.00	**-**	1.00	**-**
Yes	1.91(1.19,3.07)	**0.007**	1.64(0.92,2.93)	0.091	1.97(0.96,4.05)	0.065	1.44(0.63,3.32)	0.392
**symptom combination patterns**								
No symptom	1.00	-	1.00	-	1.00	-	1.00	-
Chronic pain only	2.66(1.22,5.83)	**0.014**	2.59(1.01,6.61)	**0.047**	0.56(0.11,2.95)	0.496	0.59(0.09,3.72)	0.571
Depressive symptoms only	2.04(0.18,23.4)	0.567	1.73(0.12,25.43)	0.689	2.25(0.18,27.96)	0.528	1.44(0.09,22.62)	0.797
Poor sleep quality only	1.75(0.93,3.28)	0.081	1.47(0.68,3.18)	0.325	1.69(0.66,4.31)	0.274	1.23(0.41,3.69)	0.712
Chronic pain and depressive symptoms	2.04(0.18,23.41)	0.567	1.08(0.03,38.92)	0.967	-	1.000	-	1.000
Poor sleep quality and depressive symptoms	5.71(1.67,19.51)	**0.005**	6.92(1.48,32.42)	**0.014**	2.42(0.73,8.02)	0.147	2.40(0.62,9.33)	0.207
Poor sleep quality and chronic pain	2.94(1.39,6.20)	**0.005**	2.48(1.02,5.98)	**0.044**	2.25(0.85,5.95)	0.102	1.45(0.47,4.54)	0.520
Poor sleep quality and chronic pain and depressive symptoms	7.34(2.26,23.84)	**0.001**	4.50(1.08,18.73)	**0.039**	1.93(0.41,9.13)	0.408	1.76(0.29,10.72)	0.542
**Symptom counts**								
0	1.00	-	1.00	-	1.00	-	1.00	-
1	1.99(1.13,3.52)	**0.018**	1.78(0.90,3.52)	0.097	1.43(0.58,3.56)	0.439	1.11(0.39,3.19)	0.849
2	3.32(1.68,6.53)	**0.001**	2.90(1.29,6.52)	**0.010**	2.25(0.90,5.62)	0.082	1.68(0.58,4.87)	0.339
3	7.34(2.26,23.84)	**0.001**	4.46(1.08,18.38)	**0.039**	1.93(0.41,9.13)	0.408	1.80(0.30,10.94)	0.525

Model 1: no adjusted model

Model 2: adjusting for age,marriage status, educational level, CIRS-G score, polypharmacy, time from admission to assessment and MMSE score

*OR* odd ratio, *CI* confidence interval

Significance difference *P* < *0.05* is shown in bold

## Discussion

This study indicated that associations of increased symptom burdens (including poor sleep quality, chronic pain and depressive symptoms) and frailty among older inpatients. More specifically, older inpatients with ≥ 2 symptoms were more prone to present frailty independent of disease burden and other related confounders, especially in those with poor sleep quality concurrent with at least one of the other two symptoms. Interaction analysis and sex stratified associations exhibited the conflicting results, and further study is required to clarify the sex-specific associations.

Consistent with existing studies, the effect of poor sleep quality, pain and depressive symptoms on physical frailty in older adults was analysed [21]. Patel and colleagues investigated six symptoms, including pain, fatigue, difficulty breathing, difficulty sleeping, depression and anxiety, and the results revealed that an increased number of symptoms was a powerful predictor for slow gait speed, poor lower extremity function and increased risk of adverse outcomes such as recurrent falls, hospitalization, disability and mortality [42]. Slow gait speed and poor lower extremity function are two critical indicators of physical frailty, which providers a good explanation for the relationship between symptom counts and frailty. Except for the impact of symptom counts on frailty, our findings also suggested that poor sleep quality concurrent with at least one of the other two symptoms was associated with frailty in older patients, with poor sleep quality combined with depressive symptoms being the most pronounced. Older adults are often concurrent with depressive symptoms and poor sleep quality, and these two symptoms interact with each other due to shared pathophysiological mechanisms [43]. Liu and colleagues showed that older adults with both poor sleep quality and depression had increased an risk of frailty compared with those with individual symptoms [44]. From the clinical treatment perspective, older patients with depressive symptoms often have concurrent with sleep disorders and increased medication burdens including sedative-hypnotic drugs and antidepressants [45]. Apart from polypharmacy, several studies have shown that taking sedative-hypnotic drugs is significantly associated with poor physical function, poor appetite and frailty [46–48]. Antidepressants, as one of the important pharmacological treatment options for clinically diagnosed depressive and anxiety disorders, are associated with frailty, which may increase the risk of adverse effects [49], falls [50], and fractures [51]. Thus, our study excluded patients with clinically diagnosed depressive or anxiety disorders who were taking antidepressants in order to avoid confounding the observed associations. Another study also found that frailty itself, in turn, worsened antidepressant treatment responses and outcomes [52, 53]. Furthermore, chronic pain, as the origin of various health hazards, was closely associated with comorbidities such as depression and sleep disorders, and further contributed to frailty and increased adverse outcomes among older adults [54]. Based on these studies, we speculated that multiple physical and psychological symptoms may have a greater impact on frailty in older inpatients.

Given that exposure to biological, behavioural and psychosocial determinants has different roles in males and females, we used the interaction analysis and sex-stratified associations to explore the sex-specific associations. However, the conflicting results were obtained. Although there was a nonsignificant effect of the interaction of sex and symptoms on frailty, sex-stratified associations showed that individual symptoms, symptom combination patterns, and symptom counts were associated with elevated risks of frailty in older male patients, but not in female patients. This may be related to the high heterogeneity of inpatients and the small sample size of individual symptom combinations. Indeed, sex differences in individual symptoms and frailty have been well reported [17, 23, 24, 26], and sex differences in multiple symptoms and frailty are rarely reported. Liu et al. indicated that sex differences were found only among difficulty maintaining sleep, difficulty initiating sleep, and frailty in the community-dwelling older adults after including two-way and three-way interaction terms, and no sex-specific associations were found among pain, depressive symptoms, and frailty [22]. Another study from the West China Health and Aging Trend study also revealed that older males with more than 5 multiple physical symptoms had a higher odds ratio of frailty [55].

There are several reasons that may be explain the sex differences in symptom combination patterns and frailty. One possible explanation is sex differences in disease severity and complexity. Older male inpatients experienced more acute conditions and complex illnesses than female inpatients [56]. Compared with females of the same age, males had a higher comorbidity load [57], such as chronic obstructive pulmonary disease, sleep-disordered breathing, sarcopenia, and sarcopenic obesity [13, 58, 59]. Acute events and multiple comorbidities increase frailty risk in older male inpatients due to multiple distressful symptoms. These symptoms may contribute to an increased risk of impaired physical function, decreased physical activity, and hospitalization associated disability, therefore contributing to incident frailty. Another explanation lies in sex differences in symptom tolerance. Females may be more concerned about their physical and psychological symptoms, but may be more tolerant of these uncomfortable symptoms. In addition, the ageing process is accompanied by changes in the composition and function of the immune system, and these changes may occur more rapidly in males than in females [60]. Disturbed sleep, depressive symptoms, and chronic pain may be involved in impacting immune function by elevating inflammatory mediators [61–63]. The dysregulation of the inflammatory process was associated with an increased risk of age-related chronic diseases and frailty [64, 65]. Therefore, more studies with a prospective design are necessary to explore sex-specific associations between multiple symptoms and frailty.

The treatment of an individual symptom can be complicated by the presence of other symptoms (e.g., pain affecting poor sleep quality by mediating depression [66]), and interventions to ameliorate these concomitant symptoms may improve subsequent frailty status. Some intervention studies have shown that these symptoms can be successfully improved simultaneously in outpatient settings [67, 68]. The adaptation of these interventions for geriatric inpatient settings should be further explored. Considering the availability of promising interventions, if comprehensively recognized and treated early, poor sleep quality, chronic pain and depressive symptoms are often reversible, but if left undertreated, these symptoms may contribute to the development of frailty and eventually lead to adverse outcomes. Hence, sex-stratified frailty risk assessment and individualized patient-centred comprehensive management should be performed in the clinical settings.

Some limitations of the current study must be noted. First, the observational cross-sectional design hinders our ability to establish casual associations and temporality, or exclude treatment-related confounding factors because of a lack of pain, sleep quality and depressive symptom changes before and after therapy. There may be complex bidirectional associations among these three symptoms themselves, and among symptoms, therapy medications, and frailty. Second, our results were from a single centre, and significant differences were observed in age, sex, smoking history, drinking history, spectrum of diseases on admission, CIRS-G score, and MMSE score between the included participants and the excluded participants, which might generate selection bias. Therefore, generalization of these results needs to be done with caution. Third, the severity of symptom patterns was not explored, and we enlarged the study samples to subdivide the severity of symptoms and further explore the associations with frailty. Fourth, the study adopted self-reported screening tools rather than clinical diagnostic measures, so the prevalence rates of poor sleep quality, chronic pain and depression in this study may have been overestimated in this study. Finally, older inpatients may represent a more heterogeneous group affected by multiple factors. The Small sample size for some symptom combination patterns (e.g., depressive symptoms only, and chronic pain and depressive symptoms) might increase some confidence intervals, and reduce the statistical power. Future studies with large samples are necessary to explore sex-specific associations between symptoms and frailty.

## Conclusions

Increased symptom burdens were associated with a higher risk of frailty in older inpatients especially in those with poor sleep quality concurrent with at least one of the other two symptoms. Our findings highlight the importance of identifying common symptom combination patterns and symptom counts in older inpatients during hospitalization, and a multidisciplinary intervention program targeting these symptom burdens is needed to reduce the risk of frailty and related adverse health outcomes.

## Supplementary Information

**Additional file 1: Table S1.** General characteristics between included patients and excluded patients.

## Data Availability

The data that support the findings of this study are available from the corresponding author upon reasonable request.

## Abbreviations

BMI: Body Mass Index
CIRS-G: Cumulative Illness Rating Scale for Geriatrics
MMSE: Mini-Mental State Examination
PSQI: Pittsburgh Sleep Quality Index
NRS: Numerical Rating Scale
GDS-15: 15-Item Geriatric Depression Scale
CFS: Clinical Frailty Scale
SD: Standard Deviation
IQR: Interquartile range
OR: Odds Ratio
CI: Confidence Interval
VIF: Variance inflation factor
