# Supplementary material for: Associations of poor sleep quality, chronic pain and depressive symptoms with frailty in older patients: is there a sex difference?
Source: BMC Geriatr. 2022 Nov 16;22:862. doi: 10.1186/s12877-022-03572-9 (PMC9667657; doi:10.1186/s12877-022-03572-9)
Supplement: Supplementary file 1 — Additional file 1: Table S1. General characteristics between included patients and excluded patients. [file 12877_2022_3572_MOESM1_ESM.docx]

**Table S1** General characteristics between included patients and excluded patients

|  | Excluded patients (n=384) | Included patients (n=540) | *P-*value |
| --- | --- | --- | --- |
| Age (mean ± SD, years) | 82.08±8.14 | 78.51±8.07 | **<0.001** |
| Age ≥80 years, n (%) | 271(70.6) | 280(51.9) | **<0.001** |
| Males, n (%) | 186(48.4) | 330(61.1) | **<0.001** |
| Educational level ≤ 6 years, n (%) | 79(20.6) | 96(17.8) | 0.285 |
| Widowed or divorced, n (%) | 73(19.0) | 127(23.5) | 0.101 |
| Current or former smoker, n (%) | 62(16.1) | 136(25.2) | **0.001** |
| Current or former drinker, n (%) | 45(11.7) | 132(24.4) | **<0.001** |
| BMI (mean ± SD, kg/m^2^) | 22.44±3.71 | 23.78±3.36 | **<0.001** |
| Main diagnosis on admission, n (%) |  |  | **<0.001** |
| Cardiovascular diseases | 50(13.1) | 113(20.9) |  |
| Peripheral vascular diseases | 101(26.4) | 222(41.1) |  |
| Nervous system diseases | 45(11.7) | 48(8.9) |  |
| Respiratory diseases | 41(10.7) | 67(12.4) |  |
| Other | 146(38.1) | 90(16.7) |  |
| CIRS-G [median (IQR), scores] | 10(7,14) | 9(7,12) | 0.013 |
| Polypharmacy (≥ 5 drugs), n (%) | 185(48.2) | 264(48.9) | 0.831 |
| MMSE (mean ± SD, scores) | 18.34±8.20 | 26.04±3.02 | **<0.001** |

*BMI* body mass index, *CIRS-G* Cumulative Illness Rating Scale for Geriatrics, *MMSE* Mini-Mental State Examination, *SD* standard deviation, *IQR* interquartile range. Significance difference *P* <0.05 is shown in bold.
